# An IMU/ODM/UWB Joint Localization System Based on Modified Cubature Kalman Filtering

**DOI:** 10.3390/s21144823

**Published:** 2021-07-15

**Authors:** Chao Tang, Chengyang He, Lihua Dou

**Affiliations:** 1 School of Automation, Beijing Institute of Technology, Beijing 100081, China; 3120170434@bit.edu.cn (C.T.); 3220180605@bit.edu.cn (C.H.); 2 Beijing Institute of Technology Chongqing Innovation Center, Chongqing 401135, China

**Keywords:** joint localization, multisensor, CKF, FKF, information fusion

## Abstract

In this article, a multisensor joint localization system is proposed based on modified cubature Kalman filtering, which aims to improve the accuracy of state estimation under a moderate computational burden in the presence of high process noise. Specifically, first, the covariance of process noise is matched based on adaptive filtering. The inertial measurement unit (IMU), odometer (ODM), and ultra-wideband (UWB) information acquired by the associated sensors is then employed to augment the system state and are fused to lower the influence of process noise. In the presented localization setting, all sensors (IMU/ODM/UWB) are set to work in parallel under the federated Kalman filter (FKF) framework, which can correct the cumulative error of the internal sensor and and can improve the computational efficiency. Two sets of numerical simulations were performed to show that the proposed method can obtain accurate state estimation with a slightly increased computational burden.

## 1. Introduction

With social and economic development, the demand for precise positioning has been increasing with the use of smart products. The global positioning system (GPS), as the most widely used method at present, is favored by its mature technology, high accuracy, and strong robustness [1], and it has been extensively employed in fields such as navigation and intelligent driving. However, it fails to provide effective positioning in most indoor scenarios, such as home services, factory logistics, cave exploration, and indoor counter-terrorism activities. In this context, it is of urgent importance to develop high-accuracy indoor positioning technology.

As an alternative to GPS, remote sensors have been widely used for indoor positioning, and they have been robustly supported by the fast development of computational devices and networks in recent years. There are a variety of remote sensor-based indoor positioning technologies, including WiFi [2,3], ZIGBEE [4], infrared [5,6], VICON [7], and Ultra-Wideband (UWB) [8,9,10]. Specifically, WiFi positioning is simple to deploy and has a low cost, but it is criticized due to its susceptibility to interference and cannot achieve high-precision positioning. In contrast, ZIGBEE has the advantages of low energy consumption, accompanied by the disadvantages of insufficient positioning accuracy. With the capacity to provide high-accuracy positioning information, infrared positioning generally fails to position hidden targets due to its reliability. In comparison, the VICON technology is featured by its high positioning accuracy and anti-interference performance, and it can be considered as a perfect indoor positioning solution except for its high cost and complicity. In this context, the UWB positioning seems to outweigh all the other counterparts due to its low cost, small size, high positioning accuracy, and strong resistance to multipath effects, all of which contribute to its wide application in multi-agent systems. Nevertheless, external sensors generally fail to provide accurate attitude information that is as equally important as position information in the multi-robot cooperative system, where attitude control is required. In this context, internal sensors, such as the inertial navigation system [11], are used to provide the attitude information of the robots in the system. Specifically, due to its mature technology and high reliability, the inertial navigation system is capable of measuring robot attitude without relying on external information. Therefore, it is not subjected to any external interference during navigating. However, this system is vulnerable to error accumulation over time, and it is not able to self-calibrate the accumulative error. As a countermeasure, internal and external sensors are integrated for the estimation of the robot’s attitude and position [12,13,14]. In these systems, the accurate attitude and position information are usually obtained by the sensors mounted inside the object (internal sensors), and external sensors in the environment are used to further calibrate this information.

Information fusion between sensors becomes an inevitable issue that has to be considered in the multisensor system. There are mainly two methods for information fusion at present, and their algorithms are, respectively, based on graph optimization [15] and filtering [16,17]. Specifically, the graph optimization-based information fusion has high accuracy, but its highly complicated calculation hinders its application in the environments with high real-time requirements. In comparison, Federated Kalman Filter (FKF)-based information fusion has been widely employed in multisensor positioning systems due to its flexible structure, strong robustness, good performance of accuracy, and easy engineering implementation [18,19].

Great progress has been recently made in the field of multisensor joint positioning. For instance, Li et al. [20] combined the location information provided by UWB and the direction location information provided by inertial measurement unit(IMU) to position coal mine robots (CMR) underground, and discussed an optimal deployment plan for UWB base stations. The final positioning accuracy was close to the most advanced lidar. Giarre et al. [21] used UWB to correct the dead reckoning results of pedestrians. Based on this joint positioning method, the positioning accuracy of pedestrians in the real environment was improved. Hu et al. [22] successfully reduced the computational load of the joint positioning system by decoupling the attitude and position information in their study using three systems (i.e., GPS, IMU, and Celestial Navigation System (CNS)) to navigate the spacecraft. Han et al. [23] managed to decrease the number of UWB nodes from three to one by calibrating and compensating GPS signals using the UWB positioning as well as fusing the information of GPS and UWB positioning using an improved particle filter based on the ant colony optimization algorithm. Using IMU sensors to supplement UWB information, Li et al. [24] successfully improved the ability of the UWB positioning system to deal with process noise by employing state enhancement, which was achieved by measuring Gaussian noise in the system speed with an accelerometer. He et al. [25] minimized the impact of drift errors by fusing the data acquired by UWB and IMU sensors in the federated Kalman filtering (FKF) framework.

Although the above-mentioned studies have made their breakthroughs, they have inherent limitations. For instance, the algorithm proposed by He et al. [25] can effectively suppress the cumulative error of the IMU data, and it has a high positioning accuracy. However, it is vulnerable to filtering divergence in the case of high process noise, which will thereby deteriorate the tracking performance. In contrast, the algorithm proposed by Li et al. [24] can effectively deal with the process noise of the system and thus has high tracking performance. However, the position information is independently provided by the external sensor, and the algorithm is based on the extended Kalman filtering (EKF) that has large linearization errors. Therefore, its positioning accuracy needs to be improved.

As a follow-up study of our previous work [25], we find that the proposed algorithm fails to effectively suppress the process noise. Accordingly, relevant research has been done in this article to pursue robust tracking, high accuracy, and high real-time performance during positioning, from the following four perspectives:

- Adaptive filtering is introduced to improve the ability of the algorithm proposed by He et al. [25] to deal with process noise.
- The expansion method proposed in Li et al. [24] is employed to expand the model, improve the tracking performance of the algorithm, and further enhance the capability of the algorithm in dealing with process noise.
- Given the increased dimension of the system caused by the expansion method, the state space decomposition method proposed in [22] is used to decompose the system state and thus reduce the side effects of the expansion method as much as possible.

After these improvements, a highly robust and accurate state estimation algorithm is obtained, though it slightly increases the computational burden. This is the main contribution of this work.

**Figure 1:**
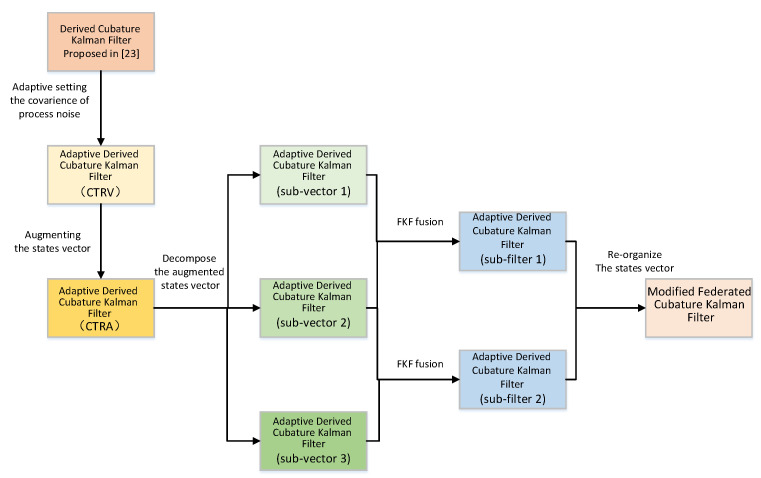
The overall structure of the algorithm.

The overall structure of the algorithm is shown in Figure 1.

The rest of this article is organized as follows. In Section 2, both the dynamic and measurement models of the system are established, accompanied by an analysis of the poor filtering effect of the process noise of the previously proposed algorithm [25]. Section 3 introduces the adaptive Kalman filtering (AKF) and improves the algorithm proposed in [25] based on this filtering method, with detailed steps illustrating the algorithm improvement. In Section 4, the state vector enhancement method proposed in [24] is introduced, and the enhanced state vector is described based on this method. Moreover, the state vector is decomposed based on the method of Hu et al. [22] to maintain the dimension of the system matrix, and thus avoid excessive computational load increase, while improving the ability of the system to deal with process noise. In Section 5, the proposed algorithm is verified by numerical simulation of the motion system. Eventually, conclusions are reached in Section 6.

## 2. System Establishment and Problem Description

### 2.1. System Model

The nonlinear discrete-time system considered in this article is

(1) $$X_{k+1}=A\left(X_{k}\right)+\omega_{k}$$

(2) $$X_{k}={\left[\begin{array}{ccccc} x & y & v & \theta & \varphi \end{array}\right]}^{T}$$

where *x* and *y* are the coordinates of the moving object, *v* is the speed of the moving object, θ is the yaw angle of the moving object, φ is the change in yaw angle over time, $\omega_{k}$ is the process noise of the system, and A(.) is a nonlinear time update. As a Constant Turn Rate and Velocity (CTRV) model, the motion model studied in this article can be expressed by the following formula:

(3) $$A\left(X_{k}\right)=A\left(\left[\begin{array}{c} x\\ y\\ v\\ \theta \\ \varphi \end{array}\right]\right)=\left[\begin{array}{c} x\\ y\\ v\\ \theta \\ \varphi \end{array}\right]+\left[\begin{array}{c} \frac{v}{\varphi }(sin\left(\theta +\varphi T\right)-sin\left(\theta \right)\\ \frac{v}{\varphi }(-cos\left(\theta +\varphi T\right)+cos\left(\theta \right)\\ 0\\ \varphi T\\ 0 \end{array}\right]$$

where *T* is the sampling time. The measurement model of the system is as follows:

(4) $$Z_{k+1}^{UWB}=H_{UWB}X_{k+1}+\upsilon_{k+1}^{uwb}=\left[\begin{array}{ccccc} 1 & 0 & 0 & 0 & 0\\ 0 & 1 & 0 & 0 & 0 \end{array}\right]X_{k+1}+\upsilon_{k+1}^{uwb}$$

(5) $$Z_{k+1}^{IMU}=H_{IMU}X_{k+1}+\upsilon_{k+1}^{imu}=\left[\begin{array}{ccccc} 1 & 0 & 0 & 0 & 0\\ 0 & 1 & 0 & 0 & 0\\ 0 & 0 & 1 & 0 & 0\\ 0 & 0 & 0 & 1 & 0\\ 0 & 0 & 0 & 0 & 1 \end{array}\right]X_{k+1}+\upsilon_{k+1}^{imu}$$

where $Z^{IMU}$ and $Z^{UWB}$ are the measurement vectors of IMU and UWB systems, respectively; $H_{IMU}$ and $H_{UWB}$ are their state transition matrices, respectively; and $\nu^{imu}$ and $\nu^{uwb}$ are independent measurement noises with Gaussian distributions, respectively. The covariances of measurement noise and process noise are

(6) $$\begin{array}{c} E\left[w_{k}w_{k}^{T}\right]=Q_{k}\\ E\left[\upsilon_{k}^{imu}\upsilon {{}_{k}^{imu}}^{T}\right]=R_{k}^{imu}\\ E\left[\upsilon_{k}^{uwb}\upsilon {{}_{k}^{uwb}}^{T}\right]=R_{k}^{uwb} \end{array}$$

where $Q_{k}$ is the covariances of process noise, and $R_{k}^{imu}$ and $R_{k}^{uwb}$ are the covaricance of measurement noise by IMU and UWB sensors, respectively.

### 2.2. Analysis of the Process Noise

In order to transplant the algorithm to the physical platform, we conducted several numerical simulation experiments using different parameters to collect data for the previously proposed Singular Value Decomposition–Federated Derived cubature Kalman Filter (SVD-FDCKF) algorithm [25]. Filtering divergence occurs in the simulation when the process noise is large. Moreover, existing filtering algorithms generally fail as the process noise exceeds certain values. Some hypotheses are put forward based on this phenomenon.

First, the covariance of the process noise (*Q*) scales with the magnitude of the process noise, and it is difficult to determine the specific value of *Q* in practical applications. Therefore, the ratio of *Q* to the covariance of measurement noise (*R*) may be too small in some cases if the value of *Q* cannot be adjusted adaptively with the magnitude of the process noise. Accordingly, in the filtering result, there might be large process noise in the moving process, thereby leading to filtering divergence.

Second, the system may not be in a uniform motion anymore due to the influence of the process noise on the system speed. The measured covariance in the iterative process might be within a reasonable range when the process noise in the experimental environment is small, because the measured residual error is small. In this case, the filtering algorithm can effectively suppress the noise. However, when the process noise in the experimental environment is set to large, the measured residual error increases, so the measured covariance grows with iteration, rendering the filter inclined to assign higher weights to the state estimation. In this way, the state estimation based on the CTRV model is no longer accurate, because the system is no longer moving at a constant speed due to the impact of noise. Therefore, filtering divergence becomes inevitable.

Given the above-mentioned issues, it is necessary to improve the existing filtering algorithms and thus enhance the tracking performance.

### 2.3. Problem Formulation

Fortunately, there have been related studies on these two issues [24,26,27]. Sage and Husa [26] proposed adaptive filtering based on the Kalman filtering in their research on the indeterminable covariance of noise. They succeeded to effectively suppress the filter divergence caused by the inaccurate noise variance. When it comes to practical application, Kownacki et al. [27] used adaptive filtering to filter the data acquired by the infrared rangefinder, which was impacted by unknown external disturbance; thus, it is difficult to determine *R*. In this way, they managed to improve the accuracy of ranging and positioning. Similar methods can also be used to suppress the imbalance between *Q* and *R* due to the large process noise, increasing the precision of the filter, thereby avoiding filtering divergence caused by excessive process noise.

In order to improve the tracking performance of the filtering algorithm for highly dynamic moving objects, Li et al. [28] used the state vector enhancement method to change the CTRV model to the CTRA model. They regarded the process noise of the speed in the state vector as the acceleration and used the accelerometer of the IMU to measure and filter it. Experimental results demonstrate that the improved algorithm can effectively suppress process noise and thus present a better performance in tracking mobile robots.

However, the measurement value of the position information in the method of Li et al. [24] is completely obtained by the UWB sensor, which can only provide decimeter-level positioning accuracy. Therefore, the state vector enhancement method will also have a certain negative impact on the stability and computational burden of the system due to the increased dimension of the state vector, although this method presents outstanding tracking performances.

To conclude, certain scientific issues need to be addressed: First, the tracking performance of the algorithm should be improved by suppressing the filtering divergence caused by process noise. Second, the dimension of the state vector should be maintained while the above improvement is achieved.

The specific solution can be divided into the following steps:

- Our previous work [25] should be improved based on the idea of adaptive filtering, so that the ratio of *Q* and *R* can still be coordinated in a high process noise environment to avoid filtering divergence.
- The model should be expanded to enhance the traceability of the system by using the state vector enhancement method [24].
- The enhanced state vector should be decomposed according to the measurement vector of each sensor [28] to reduce the dimension of the system and thus improve the measurement accuracy of the system.

## 3. Adaptive SVD-DCKF Filtering

### 3.1. Preliminary Work

As shown in Section 2.1, the system features a nonlinear system state equation and a linear measurement equation. In this case, a redundant calculation occurs, and accuracy will be lowered due to linearization errors, if the unscented Kalman filter (UKF) and cubature Kalman filter (CKF) and other nonlinear Kalman filtering algorithms are used for filtering. As inspired by the DUKF algorithm in the literature [29], we propose the Derived Cubature Kalman Filter (DCKF), which reduces the computational burden of the system and eliminates some unnecessary linearization errors.

Moreover, inspired by the random stability analysis in the literature [30], we further develop the SVD-DCKF algorithm [25] based on the DCKF algorithm. This SVD-DCKF algorithm can replace the traditional Cholesky decomposition with the SVD decomposition during the decomposition of the estimated covariance of the system (*P*). In this way, the constraining conditions are expanded from positive definite variance to positive semi-definite, so that the algorithm no longer needs the positive definite auxiliary matrix (δP) and thus eliminates the minor errors caused by the auxiliary matrix. The specific steps of the SVD-DCKF algorithm have been described in detail in the literature [25] and thus are not repeated here.

In addition, in order to suppress the cumulative error of the IMU, we adopted the UWB to continuously calibrate the IMU under the FKF framework. As the result, the effectiveness of this method [25] is demonstrated through numerical simulations.

However, the SVD-DCKF algorithm is found to have a deteriorating and even divergent filtering performance under certain conditions with different process and measurement noises. Accordingly, this study aims to improve the SVD-DCKF algorithm based on the adaptive filtering method.

### 3.2. Adaptive Filtering

The *Q* and *R* have direct impacts on the performance of Kalman filtering algorithms [27]. According to the calculation equation of the Kalman gain (*K*), *K* is proportional to *Q* and inversely proportional to *R*. Therefore, when *Q* is relatively large, the process noise has great interference with respect to the state prediction, and accordingly, *K* is relatively large, which will increase the weight of the measurement equation in the filter and thus modify the state estimation with more measurement values. In this context, an undersized *Q* might be responsible for the filtering divergence that occurs in the application of the algorithm proposed in [25] in a high process noise environment. However, an excessively large *Q* can reduce the filtering accuracy [26]. Moreover, it is generally very difficult to accurately simulate the system noise to determine the optimal and accurate value of *Q* in practical applications. Consequently, it is challenging to achieve the best performance of the filter in engineering by setting *Q* directly at the initial moment according to the prior value. Therefore, adaptive filtering technology is often used to adaptively modify *Q*.

Considering the system shown in Section 2.1, the residual error ($e_{k}$) should be first calculated during the application of the adaptive filtering:

(7) $$e_{k}=X_{k}-X_{k|k-1}$$

where $X_{k}$ is the state estimation of the system at time *k*, while $X_{k|k-1}$ is a one-step prediction of the system state at time *k* based on the system state at time k−1.

The correction matrix of the process noise covariance at time *k* ($Q_{k}^{*}$) should then be calculated based on the residual error of the system:

(8) $$Q_{k}^{*}=e_{k}e_{k}^{T}+P_{k|k-1}-P_{k}-Q_{k-1}$$

where $P_{k|k-1}$ is a one-step prediction of the system error variance at time *k* based on the system error variance at time k−1, while $Q_{k-1}$ is the process noise covariance estimation at time k−1.

Subsequently, a low-pass filter should be used to modify the matrix $Q_{k}^{*}$ and the noise covariance $Q_{k-1}$ at the previous moment, yielding the process noise covariance estimate at time *k* ($Q_{k}$):

(9) $$Q_{k}=Q_{k-1}+\frac{\left(Q_{k}^{*}-Q_{k-1}\right)}{M}$$

where *M* is the window width, and its value can be obtained by the following formula:

(10) $$\left\{\begin{array}{cc} M=1, & d\geq 1\\ M=k, & d\leq 0\\ M=kd, & 0<d<1 \end{array}\right.$$

where *d* can be obtained by the residual value formula:

(11) $$d=e_{k}^{T}E\left[e_{k}e_{k}^{T}\right]e_{k}$$

When d>1, the statistical characteristics of the process noise are not clear, the system cannot get the correct state prediction, and the minimum value should be adopted. When d<0, the statistical characteristics of the process noise are clear, so the prediction of the system’s state equation is relatively accurate, and the maximum value should be adopted. When 0<d<1, the middle value should be adopted according to the ratio to ensure that *Q* is in the right state.

### 3.3. The SVD-ADCKF Algorithm Based on Adaptive Filtering

The SVD-DCKF algorithm proposed in [25] is improved based on the adaptive filtering method described in the previous section. It is able to adaptively adjust *Q* under different process noise environments, and this improved algorithm is termed the Singular Value Decomposition-Adaptive Derive Cubature Kalman Filtering (SVD-ADCKF). The specific steps of the algorithm are shown in Algorithm 1. It can be seen from Algorithm 1 that the proposed algorithm is basically similar to the original CKF, but a time update step for the process noise covariance is added, which allows it to adaptively approach the true value as the number of iterations increases.


**Algorithm 1. The specific steps of SVD-ADCKF.**
**      Input:**$X_{k}$, $P_{k}$, $Q_{k}$**      Output:**$X_{k+1}$, $P_{k+1}$, $Q_{k+1}$
**      Nonlinear time update**
      1. Initialize state estimation $X_{k}$ and covariance estimation $P_{k}$;      2. Perform SVD decomposition on $P_{k}$ to obtain $S_{k}$;      3. Calculate the cubature point $X_{k}^{j}$ according to the equation: $\left\{\begin{array}{cc} X_{k}^{j}=X_{k}+S_{k}I_{j}\sqrt{n} & ,j=1,\ldots ,n\\ X_{k}^{j}=X_{k}-S_{k}I_{j}\sqrt{n} & ,j=n+1,\ldots ,2n \end{array}\right.$;      4. Calculate the one-step prediction of each cubature point ($X_{k+1|k}^{j}$) based on the state equation and   the process noise covariance ($Q_{k}$);      5. Combine each cubature point to obtain the linear approximation time update $X_{k+1|k}$:
$$X_{k+1|k}=\sum_{j=1}^{2n}\omega_{j}X_{k+1|k}^{j}$$
quad where *n* is system order; $\omega_{j}$ is the weight of each cubature point;      6. Calculate the time update of the linearly approximated error covariance $P_{k+1|k}$:
$$P_{k+1|k}=\sum_{j=1}^{2n}\omega_{j}(X_{k+1|k}^{}-X_{k+1|k}^{}){\left(X_{k+1|k}^{}-X_{k+1|k}^{}\right)}^{T};$$
**      Linear measurement update**
      7. Obtain the linear prediction value of the measurement ($Z_{k+1|k}$) based on the time update of   the measurement equation to the linear approximation;      8. Calculate Kalman gain *K* based on the formula: $K=P_{k+1|k}H^{T}{\left(HP_{k+1|k}H^{T}+R_{k}\right)}^{-1}$;      9. Update the system state based on the Kalman gain: $X_{k+1}=X_{k+1|k}+K\left(Z_{k+1}-Z_{k+1|k}\right)$;      10. Update the system error covariance based on the Kalman gain: $P_{k+1}=P_{k+1|k}-KHP_{k+1|k}$;
**      Process noise covariance update**
      11. Calculate the residual error: $e_{k+1}=X_{k+1}-X_{k+1|k}$;      12. Calculate the correction matrix: $Q_{k+1}^{*}=e_{k+1}e_{k+1}^{T}+P_{k+1|k}-P_{k+1}-Q_{k}$;      13. Calculate the window width M based on Formulas (10) and (11);      14. Correct the process noise covariance: $Q_{k+1}=Q_{k}+\frac{\left(Q_{k+1}^{*}-Q_{k}\right)}{M}$;      15. Output $X_{k+1}$, $P_{k+1}$, $Q_{k+1}$ as the initial value for the next moment, and repeat Steps 1–14.**      End**.

## 4. Improvement in the Tracking Performance

### 4.1. System Model after the State Vector Enhancement

In the work of Li et al. [24], the noise of the speed of the system is regarded as the acceleration of the system, and the acceleration in the X-axis and Y-axis directions is introduced into the state vector of the system through state vector enhancement and then measured by the IMU sensor. Subsequently, they filtered the augmented system state based on the estimation of the system state and the measured value of the sensor, the results of which demonstrate the improved tracking performance.

The system state vector defined by Equation (2) can be correspondingly rewritten as

(12) $$X_{k}={\left[\begin{array}{cccccc} x & y & v & a & \theta & \varphi \end{array}\right]}^{T}$$

where *a* is the acceleration of the system.

Meanwhile, the time update equation of the nonlinear discrete system shown in Equation (3) can be defined as

(13) $$A\left(X_{k}\right)=A\left(\left[\begin{array}{c} x\\ y\\ v\\ a\\ \theta \\ \varphi \end{array}\right]\right)=\left[\begin{array}{c} x\\ y\\ v\\ a\\ \theta \\ \varphi \end{array}\right]+\left[\begin{array}{c} \Delta x\\ \Delta y\\ aT\\ 0\\ \varphi T\\ 0 \end{array}\right]$$

where *T* is the sampling time, while Δx and Δy are the displacements in the *x* and *y* directions, respectively, which can be expressed as

(14) $$\Delta x=\frac{\left(v+aT)\sin(\theta +\varphi T)-v\sin(\theta \right)}{\varphi }+\frac{a}{\varphi^{2}}\left[\cos\left(\theta +\varphi T\right)-\cos\left(\theta \right)\right]$$

(15) $$\Delta y=\frac{\left(v+aT)\cos(\theta +\varphi T)-v\cos(\theta \right)}{\varphi }+\frac{a}{\varphi^{2}}\left[\sin\left(\theta +\varphi T\right)-\sin\left(\theta \right)\right]$$

The measurement model used in the new sixth-order CTRA system is defined as

(16) $$Z_{k+1}^{UWB}=H_{UWB}X_{k+1}+\upsilon_{k+1}^{uwb}=\left[\begin{array}{cccccc} 1 & 0 & 0 & 0 & 0 & 0\\ 0 & 1 & 0 & 0 & 0 & 0\\ 0 & 0 & 1 & 0 & 0 & 0 \end{array}\right]X_{k+1}+\upsilon_{k+1}^{uwb}$$

(17) $$Z_{k+1}^{IMU}=H_{IMU}X_{k+1}+\upsilon_{k+1}^{imu}=\left[\begin{array}{cccccc} 0 & 0 & 1 & 0 & 0 & 0\\ 0 & 0 & 0 & 1 & 0 & 0\\ 0 & 0 & 0 & 0 & 1 & 0\\ 0 & 0 & 0 & 0 & 0 & 1 \end{array}\right]X_{k+1}+\upsilon_{k+1}^{imu}$$

Moreover, an odometer(ODM) is introduced as an auxiliary internal sensor in the revised system, and its measurement model can be expressed as

(18) $$Z_{k+1}^{\mathrm{ODM}}=H_{ODM}X_{k+1}+\upsilon_{k+1}^{odm}=\left[\begin{array}{cccccc} 1 & 0 & 0 & 0 & 0 & 0\\ 0 & 1 & 0 & 0 & 0 & 0 \end{array}\right]X_{k+1}+\upsilon_{k+1}^{odm}$$

### 4.2. Accuracy of the Augmented System Model

A relatively large cumulative error is inevitable in the augmented system model as the dead reckoning of the IMU sensor is based on the quadratic integration of the data recorded by the accelerometer. As a countermeasure, the IMU sensor in the modified system is now used to only measure the velocity, acceleration, yaw angle, and yaw angle movement change of the mobile robot, while the position information of the system is provided by the odometer that directly calculates dead reckoning based on the number of turns of the wheel. In this context, the acceleration, yaw angle, and yaw angle movement change of the system are directly measured by the accelerometer, magnetometer, and gyroscope, respectively, which reduced the cumulative error. The accelerometer in the IMU measures the specific force other than the gravity received by the object, calculates it as acceleration, and finally outputs the acceleration. There are still certain cumulative errors in one integral calculation of the system velocity by the accelerometer and one integral calculation of the position information by the odometer. However, these cumulative errors are relatively smaller than that of the system introduced in Section 2.1, which requires quadratic integration. Moreover, the position and velocity information of the system that is provided by the external UWB sensor is used to calibrate the cumulative error of the internal sensor.

### 4.3. Simplification of the State Vector

Although the state vector enhancement method, when used, can effectively improve the tracking performance of the system and suppress the process noise, the dimension of the state vector will increase accordingly. In this context, the increased dimension of the state vector is accompanied by the increased computational burden of UKF and CKF algorithms, although the computational accuracy and stability are rarely influenced [28,31]. In this study, the state vector disassembly method [22] and the state vector enhancement method [24] are integrated to minimize the side effects caused by the state vector enhancement method.

In some integrated IMU/CNS systems, the CNS can output accurate attitude information and relatively accurate position information, but it cannot perform high-precision calculations of velocity information [22]. As a result, the errors in the system’s error covariance matrix corresponding to the estimated velocity error continually grows. Therefore, it can be inferred that there is an extremely weak correlation among attitude, velocity, and position error information in the local state estimation output by the subsystems of the integrated IMU/CNS system [22]. Given this, Hu et al. [22] disassemble the state vector into three sub-vectors in the integrated IMU/GPS/CNS navigation system and thereby successfully reduce the order of the state vector, contributing to a lowered computational burden of the sub-filter.

Moreover, we find that there is only a one-way strong correlation between position information, velocity information, and attitude information in the CTRA system. More specifically, the accurate estimation of position information depends on the velocity information and attitude information, whereas the accurate estimation of velocity information and attitude information rarely depends on the position information. Accordingly, this study simplifies the state vector of the IMU sub-filter based on the fact that the IMU sensor only provides the velocity information and attitude information in this research, and the simplified sub-vector is as follows:

(19) $$X_{k}^{IMU}={\left[\begin{array}{cccc} v & a & \theta & \phi \end{array}\right]}^{T}$$

The new time update equation and the nonlinear time update function are defined as follows:

(20) $$X_{k}^{IMU}=A^{IMU}\left(X_{k}^{IMU}\right)+w_{k}^{IMU}$$

(21) $$A^{IMU}\left(X_{k}^{IMU}\right)=A\left(\left[\begin{array}{c} v\\ a\\ \theta \\ \phi \end{array}\right]\right)=\left[\begin{array}{c} v\\ a\\ \theta \\ \phi \end{array}\right]+\left[\begin{array}{c} aT\\ 0\\ \phi T\\ 0 \end{array}\right]$$

It can be seen that the state vector of the simplified IMU sub-filter is a fourth-order system, and the measurement model for the new fourth-order system is

(22) $$Z_{k+1}^{IMU}=H_{IMU}X_{k+1}^{IMU}+\upsilon_{k+1}^{imu}=\left[\begin{array}{cccc} 1 & 0 & 0 & 0\\ 0 & 1 & 0 & 0\\ 0 & 0 & 1 & 0\\ 0 & 0 & 0 & 1 \end{array}\right]X_{k+1}+\upsilon_{k+1}^{imu}$$

After simplification, the state vectors of the internal sensors of the new system are composed of the sixth-order ODM sensor and the fourth-order IMU sensor. In comparison, the state vectors of internal sensors in the original system before the enhancement are composed of the fifth-order ODM sensor and the fifth-order IMU sensor, whereas those in the CTRA system after the enhancement are composed of the sixth-order ODM sensor and the sixth-order IMU sensor. The computational complexity of these three systems will be analyzed in the next subsection.

### 4.4. Computational Complexity Analysis

Efforts were made in this study to analyze the computational complexity of the filtering operation on the internal sensors based on the “Singular Value Decomposition-Derived Cubature Kalman filter” (SVD-DCKF) algorithm [25]. We refer to each multiplication or addition operation as a flop, and the total number of flops represents the computational complexity of the algorithm (Table 1).

As shown in Table 2, the augmented system requires 3054 more flops (43.42%) than the original system in each iteration, while the simplified augmented system requires just 550 more flops (12.11%) than the original system. Therefore, it can be concluded that the simplified augmentation system successfully reduced the computational burden of the original augmentation system while maintaining a strong tracking performance.

### 4.5. Fusion and Reorganization of Sub-States

Disregarding their ability to provide accurate position, velocity, and attitude information, internal sensors cannot calibrate the cumulative errors that occur during the calculation. In contrast, external sensors usually have low accuracy, but they are not subjected to cumulative errors. Therefore, the fusion based on the FKF framework can make the external sensor calibrate the internal sensor and improve the positioning accuracy.

**Figure 2:**
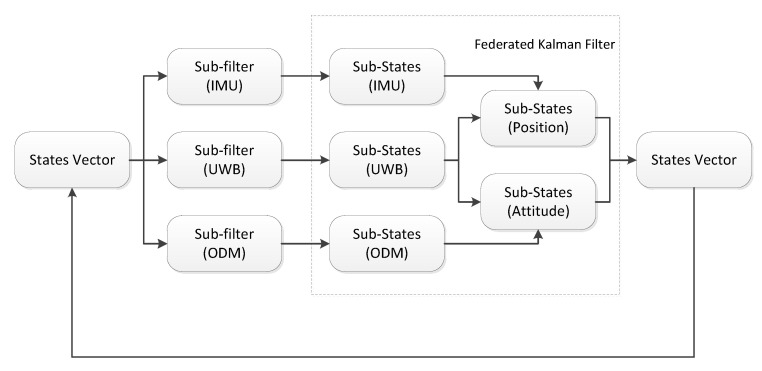
The overall structure of the system.

As shown in Figure 2, three sub-state vectors and their covariance matrices can be obtained after three different sensors are filtered by the sub-filter. Specifically, three sub-state vectors are expressed as

(23) $$\begin{array}{c} X_{k}^{UWB}={\left[\begin{array}{cccccc} x_{uwb} & y_{uwb} & v_{uwb} & a_{uwb} & \theta_{uwb} & \phi_{uwb} \end{array}\right]}^{T}\\ X_{k}^{IMU}={\left[\begin{array}{cccc} v_{imu} & a_{imu} & \theta_{imu} & \phi_{imu} \end{array}\right]}^{T}\\ X_{k}^{ODM}={\left[\begin{array}{cccccc} x_{odm} & y_{odm} & v_{odm} & a_{odm} & \theta_{odm} & \phi_{odm} \end{array}\right]}^{T} \end{array}$$

As has been described in Section 3.1, the UWB sensor only measures position information and velocity information, the ODM sensor only observes position information, and the IMU sensor only records accelerate information for the information fusion. Therefore, redundant information in the sub-vectors can be eliminated, and the three sub-state vectors can be rewritten as

(24) $$\begin{array}{c} X_{k|k}^{UWB}={\left[\begin{array}{ccc} x_{uwb} & y_{uwb} & v_{uwb} \end{array}\right]}^{T}\\ X_{k|k}^{IMU}=\left[v_{imu}\right]\\ X_{k|k}^{ODM}={\left[\begin{array}{cc} x_{odm} & y_{odm} \end{array}\right]}^{T} \end{array}$$

The covariance matrices of UWB and ODM sensors are both of the sixth-order, while that of the IMU sensor is of the fourth-order. Therefore, the covariance matrix of each sub-vector should also be simplified according to the simplified sub-state vectors. The simplified covariance matrices are as follows:

(25) $$P_{k|k}^{UWB}=\left[\begin{array}{ccc} P_{k,11}^{UWB} & P_{k,12}^{UWB} & P_{k,13}^{UWB}\\ P_{k,21}^{UWB} & P_{k,22}^{UWB} & P_{k,23}^{UWB}\\ P_{k,33}^{UWB} & P_{k,33}^{UWB} & P_{k,33}^{UWB} \end{array}\right]$$

(26) $$P_{k|k}^{IMU}=\left[P_{k,33}^{IMU}\right]$$

(27) $$P_{k|k}^{\mathrm{ODM}}=\left[\begin{array}{cc} P_{k,11}^{\mathrm{ODM}} & P_{k,12}^{\mathrm{ODM}}\\ P_{k,21}^{\mathrm{ODM}} & P_{k,22}^{\mathrm{ODM}} \end{array}\right]$$

where $P_{k}^{UWB},P_{k}^{IMU}$, and $P_{k}^{ODM}$ are the covariance matrices of the corresponding sub-state vectors, while $P_{k|k}^{UWB},P_{k|k}^{IMU}$, and $P_{k|k}^{ODM}$ are the covariance matrices of the simplified sub-state vectors by eliminating redundant information. These covariance matrices are then fused based on the FKF framework:

(28) $$P_{k}^{Position}={\left({\left[\begin{array}{cc} P_{k,11}^{UWB} & P_{k,12}^{UWB}\\ P_{k,21}^{UWB} & P_{k,22}^{UWB} \end{array}\right]}^{-1}+P_{k|k}^{ODM-1}\right)}^{-1}$$

(29) $$P_{k}^{Velocity}={\left(P_{k,33}^{UWB-1}+P_{k|k}^{IMU-1}\right)}^{-1}$$

where $P_{k}^{Position}$ is the covariance of the position information after fusion at time *k*, while $P_{k}^{Velocity}$ is the covariance of the velocity information after fusion at time *k*. Subsequently, the sub-state vectors of the fused position information and velocity information are calculated based on the fused covariance:

(30) $$X_{k}^{Position}=P_{k}^{Position}\left({\left[\begin{array}{cc} P_{k,11}^{UWB} & P_{k,12}^{UWB}\\ P_{k,21}^{UWB} & P_{k,22}^{UWB} \end{array}\right]}^{-1}\left[\begin{array}{c} X_{k|k,11}^{UWB}\\ X_{k|k,21}^{UWB} \end{array}\right]+P_{k|k}^{ODM-1}X_{k|k}^{ODM}\right)$$

(31) $$X_{k}^{Velocity}=P_{k}^{velocity}\left(P_{k|k}^{IMU-1}X_{k|k}^{IMU}+P_{k,33}^{\mathrm{UWB}-1}X_{k,31}^{\mathrm{UWB}}\right)$$

where $X_{k}^{Position}$ and $X_{k}^{Velocity}$ are the sub-state vectors of the fused position information and velocity information, respectively. At this time, we can obtain the sub-state vector of attitude by combining the remaining information in the sub-state vector of the IMU sensor, which is expressed as

(32) $$X_{k}^{Attitute}={\left[\begin{array}{cccc} X_{k}^{Velocity} & a_{imu} & \theta_{imu} & \phi_{imu} \end{array}\right]}^{T}$$

Finally, as shown in Figure 2, sub-vectors of the position information and the attitude information are recombined to form a new state vector:

(33) $$X_{k}=\left[\begin{array}{c} X_{k}^{Position}\\ X_{k}^{Attitute} \end{array}\right]$$

Similarly, by extracting the covariance components corresponding to each state component and recombining, the new covariance can be defined as

(34) $$P_{k}=\left[\begin{array}{ccccc} P_{k}^{Position} & & & & \\ & P_{k}^{Velocity} & & & \\ & & P_{k,22}^{imu} & & \\ & & & P_{k,33}^{imu} & \\ & & & & P_{k,44}^{imu} \end{array}\right]$$

External sensors have been demonstrated to be effective in calibrating the cumulative error of the inertial navigation system based on the FKF framework [25]. Similarly, the odometer should be theoretically able to be calibrated using the same method. Moreover, the data calibrated in this study are obtained by only one integral operation, so their accuracy should be theoretically higher than those of the position information in He et al. [25] (which are obtained by quadratic integral operations). Therefore, it is reasonable to believe that the IMU/UWB/ODM co-location system should have better accuracy than the IMU/UWB system, and relevant numerical simulation will be detailed in Section 5.

Eventually, the proposed algorithm in this study, namely, the Modified Federated Cubature Kalman Filter (MFCKF), is capable of accurately estimating the attitude information of the system after decomposition and state reorganization. Moreover, it has a high real-time tracking performance. The specific steps of the proposed algorithm are detailed in Algorithm 2.


**Algorithm 2. Specific steps of the MFCKF.**
**      Input:**$X_{k}$, $P_{k}$, $Q_{k}$**      Output:**$X_{k}+1$, $P_{k}+1$, $Q_{k+1}$      1. Decompose $X_{k}$ and $P_{k}$ and obtain sub-states (i.e., $X_{k}^{imu}$, $X_{k}^{uwb}$, and $X_{k}^{odm}$)   and their covariance (i.e., $P_{k}^{imu}$, $P_{k}^{uwb}$, and $P_{k}^{odm}$);      2. Filter the sub-filters based on the sub-states and sub-covariances as well as the measured values and          obtain the sub-states and sub-covariances at time k+1 ($X_{k+1}^{imu}$, $X_{k+1}^{odm}$, $X_{k+1}^{uwb}$ and $P_{k+1}^{imu}$, $P_{k+1}^{odm}$, $P_{k+1}^{uwb}$, $Q_{k+1}$)   (based on the filter shown in Algorithm 1);      3. Eliminate redundant information and get sub-states for fusion (i.e., $X_{k+1}^{imu}$, $X_{k+1}^{uwb}$, and $X_{k+1}^{odm}$)          and their variances (i.e., $P_{k+1}^{imu}$, $P_{k+1}^{uwb}$, and $P_{k+1}^{odm}$);      4. Fuse the sub-states and covariance based on the FKF framework and thus obtain $X_{k+1}^{Velocity}$ and $X_{k+1}^{Position}$          as well as their covariance $P_{k+1}^{Velocity}$ and $P_{k+1}^{Position}$;      5. Recombine $X_{k+1}^{imu}$, $X_{k+1}^{Velocity}$ and $X_{k+1}^{Position}$ to obtain the final status update $X_{k+1}$;      6. Recombine $P_{k+1}^{imu}$, $P_{k+1}^{Velocity}$ and $P_{k+1}^{Position}$ to obtain the final status update $P_{k+1}$;      7. Output $X_{k+1}$, $P_{k+1}$, $Q_{k+1}$ as the initial value for the next moment, and repeat Steps 1–6.**      End**.

## 5. Simulation

Simulations were performed on an x86 PC using MATLAB 2019a with Intel Core i5 7500 CPU and 16 GB memory to fully evaluate the performance of the proposed algorithm. The simulation program used was MATLAB 2019a.

Two sets of simulations were conducted. The first set mainly aims to test the relative performance of the improved SVD-ADCKF algorithm to the SVD-DCKF algorithm. Both of these are based on the FKF framework to fuse multisensor information, thereby they are called SVD-FDCKF and SVD-FADCKF in the results of simulations. Specifically, this set of simulations evaluates the performances of these two algorithms in different process noise environments to verify the adaptive function of the improved algorithm, with the main focus on the accuracy of the state estimation, the Standard Deviation, the error covariance, and the convergence algebra of the algorithm. The second set of simulations introduces the MFCKF algorithm proposed in Section 4 and aims to verify the capacity of the state enhancement method in more efficiently suppressing the process noise. In this set of simulations, the calculation time was also added based on the first set of simulations to qualitatively analyze whether the algorithm after state decomposition will bring a great additional calculation burden.

**Figure 3:**
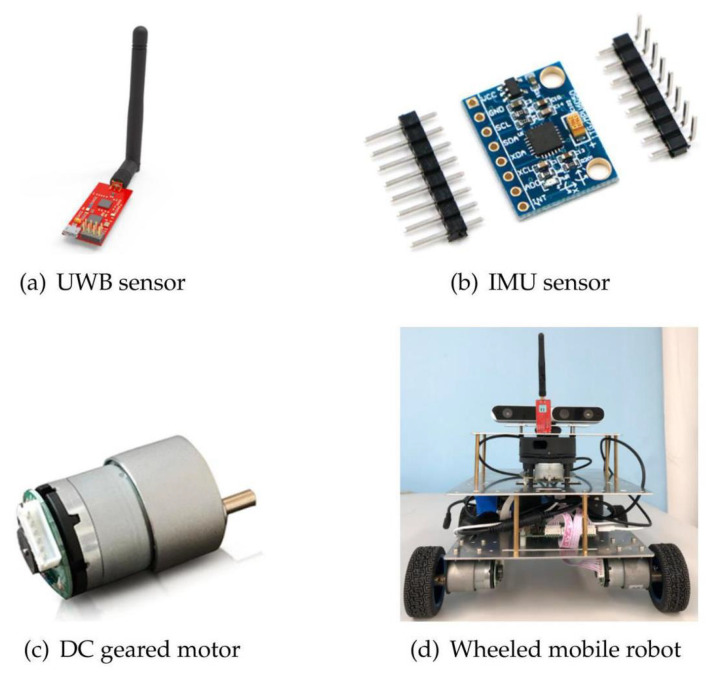
The sensors and the mobile robot involved in this article: (**a**) The Ultra-Wide Band (UWB) sensors (YCHIOT, Wenzhou, Zhejiang, China), which are commercial products provided by the company of YCHIOT, with the module of Mini3s; (**b**) the spatial motion sensor chip MPU9150 as the IMU sensor (Digi-Key Electronics, Thief River Falls, Minnesota, United States); (**c**) the odometer84 constructed by the DC gear motors MG513 with an encoder (Fenghua Transmission, Kunshan, Jiangsu, China); and (**d**) the wheeled mobile robot platform (Ruiqu Technology, Foshan, Guangdong, China) that realizes the precise localization by carrying the above sensors.

The models for numerical simulations are described in Section 2.2 and Section 4.1. Uncorrelated Gaussian white noise is added as the process noise of the system, and the noise of the position information conforms to a normal distribution. Specific parameters of simulations as shown in Table 2. The estimation covariance will iteratively converge in the filter, therefore the iterative initial value of the estimation covariance is a non-optimal value given based on experience. To initialize 1500 Monte Carlo simulations, the iterative initial value of the state vector in each simulation is equal to the true initial state plus a random component extracted from a Gaussian distribution with zero mean and variance equal to the initial covariance of the filter. In order to make it easier to transplant the algorithm to a physical platform for testing in the future, the measurement noise of the simulation is close to the parameters of the physical components that are expected to be used (see Figure 3; the actual parameters of simulations are subject to Table 2). In this context, the results of the numerical simulations can be as close as possible to the actual situation. (For technical reasons, the experiment is still at the simulation phase currently.)

**Figure 4:**
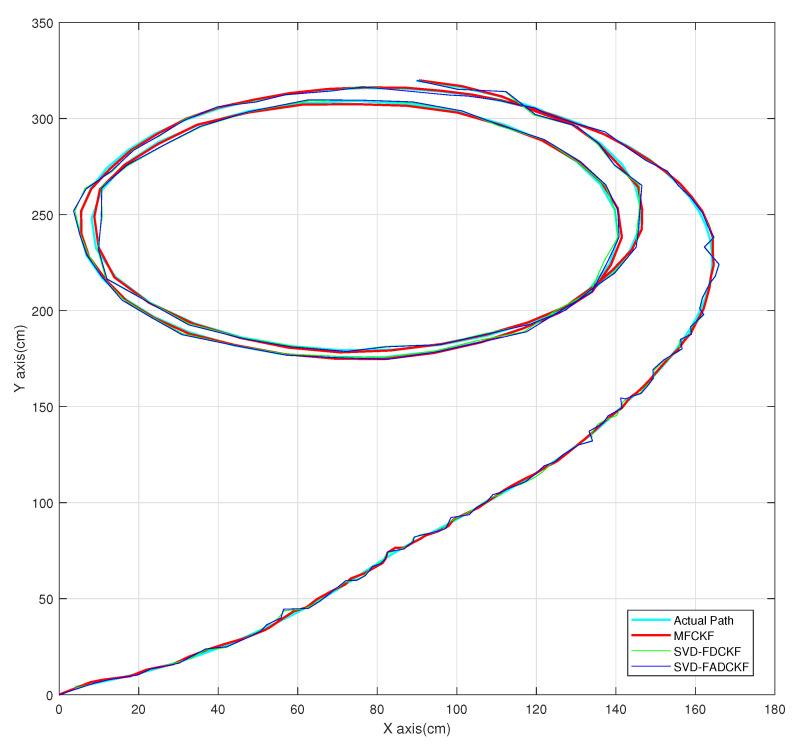
Simulation paths of different algorithms.

Figure 4 shows the simulation paths of different algorithms. More detailed analyses are presented in Section 5.1 and Section 5.2.

### 5.1. Simulation on the SVD-FADCKF

**Figure 5:**
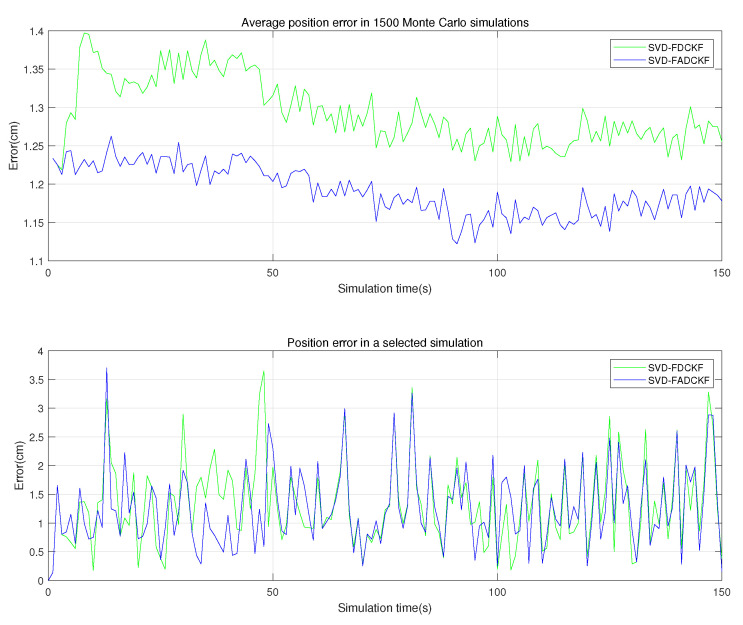
Positioning errors of the two algorithms.

**Figure 6:**
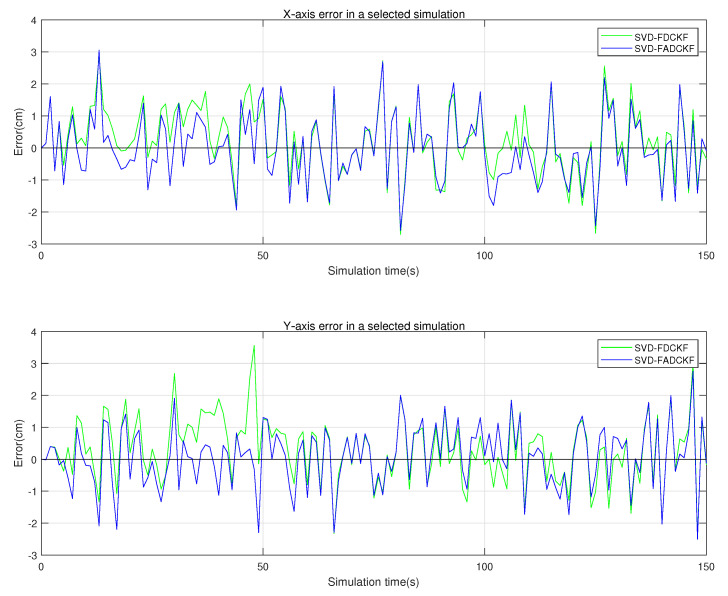
*X*-axis and *Y*-axis errors of the two algorithms in selected simulations.

The purpose of this set of simulations is to verify the performance improvement of the SVD-ADCKF algorithm compared to the original algorithm (SVD-DCKF). Based on the algorithm proposed in Section 3, filtering is implemented in the IMU and UWB systems, respectively, and fusion is achieved under the FKF framework (SVD-FADCKF and SVD-FDCKF). Meanwhile, the estimation error of the state vector is recorded and evaluated (Figure 5 and Figure 6).

As shown in Figure 5, the improved algorithm with the introduced adaptive factor (SVD-ADCKF) has an obviously lower average positioning error than the original algorithm (SVD-DCKF). Table 3 shows that the average positioning error of SVD-ADCKF is ~7.89% lower than that of SVD-DCKF.

As shown in Figure 5 and Figure 6, the two algorithms have similar positioning errors in most cases, but SVD-ADCKF performs better when the error fluctuates greatly. This may be attributed to the adaptive adjustment of the process noise covariance by SVD-ADCKF when the time update of the system is inaccurate. In this way, the weight of observations in the state estimation is increased, thereby reducing the overall error.

### 5.2. Simulation on the MFCKF

**Figure 7:**
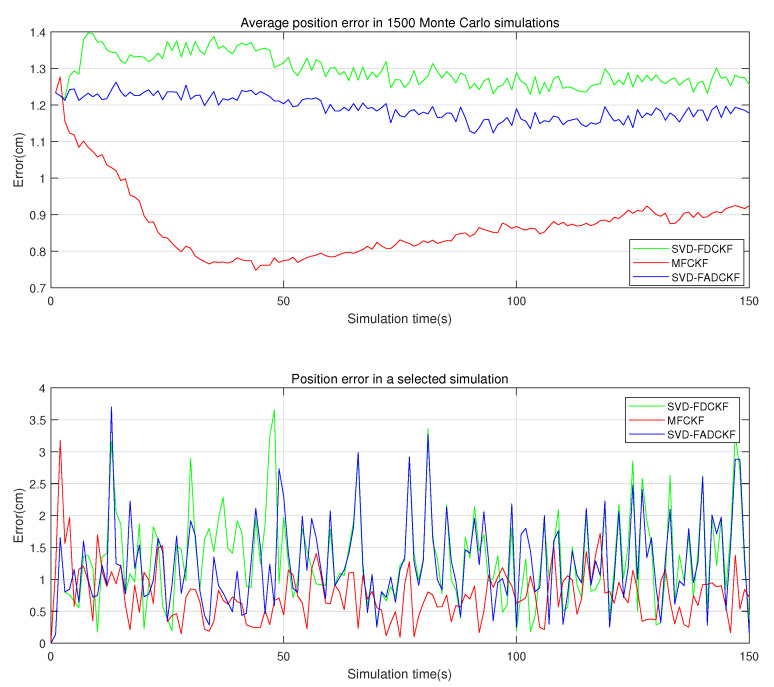
Positioning errors of the three algorithms.

**Figure 8:**
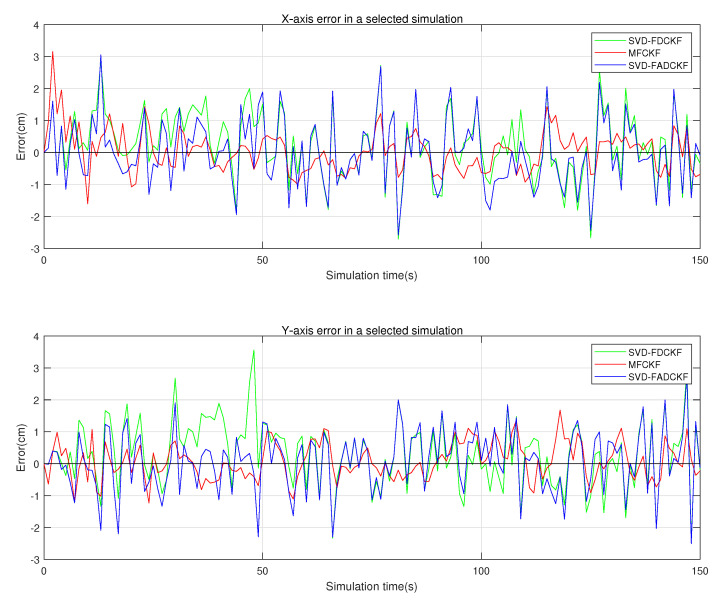
*X*-axis and *Y*-axis errors of the three algorithms in selected simulations.

**Figure 9:**
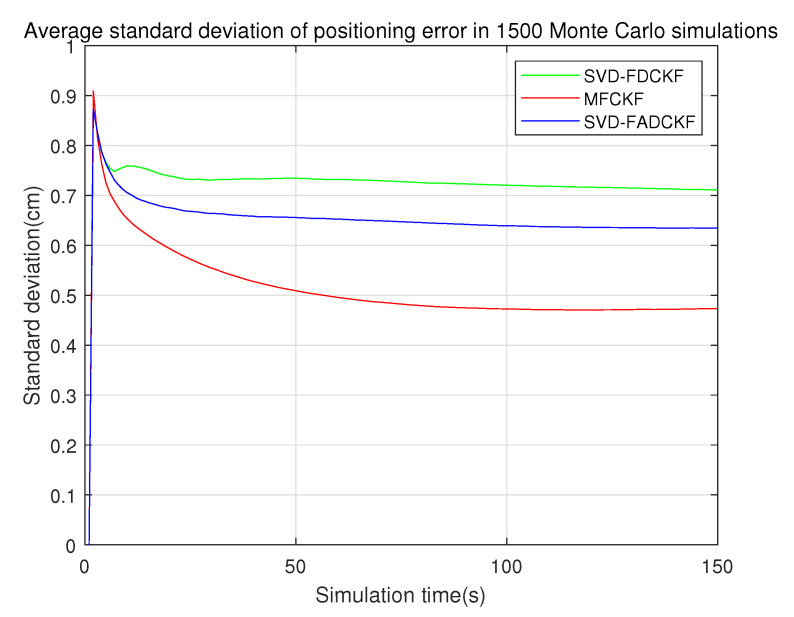
Average standard deviation of positioning error in 1500 Monte Carlo experiments (3 algorithms).

This set of simulations mainly aims to verify the performance improvement of the MFCKF algorithm relative to the original algorithm (SVD-FDCKF) and the adaptive algorithm (SVD-FADCKF). The main experimental environment is the same as that in Section 5.1, while the difference is the addition of the algorithm proposed in Section 4 as the experimental object, which can record and evaluate the estimation error and standard deviation of the state vector. The simulation results are shown in Figure 7, Figure 8 and Figure 9.

As is shown in Figure 7, the MFCKF not only has better performance than the original algorithm, but also has a lower positioning error than the adaptive algorithm proposed in Section 3 (SVD-FADCKF). As can be seen in Table 4, the average positioning error of the improved algorithm after state enhancement is about 32.76% lower than that of the original algorithm (SVD-FDCKF).

As shown in Figure 7 and Figure 8, the error curve becomes smoother after the enhancement of the state vector and the addition of the new state information. Therefore, it can be inferred that the state enhancement method can improve the algorithm in terms of tracking the state of moving bodies. However, it is also noticed that the improved algorithm deteriorates in terms of initial overshoot and dynamic response performance, which may be caused by the increase in the order of the state vector. More efforts are needed to further address this issue.

Figure 9 shows the positioning standard deviation of each iteration in the 1500 Monte Carlo experiments. It is clear from the figure that the standard deviation of MFCKF is better than the other two algorithms.

**Figure 10:**
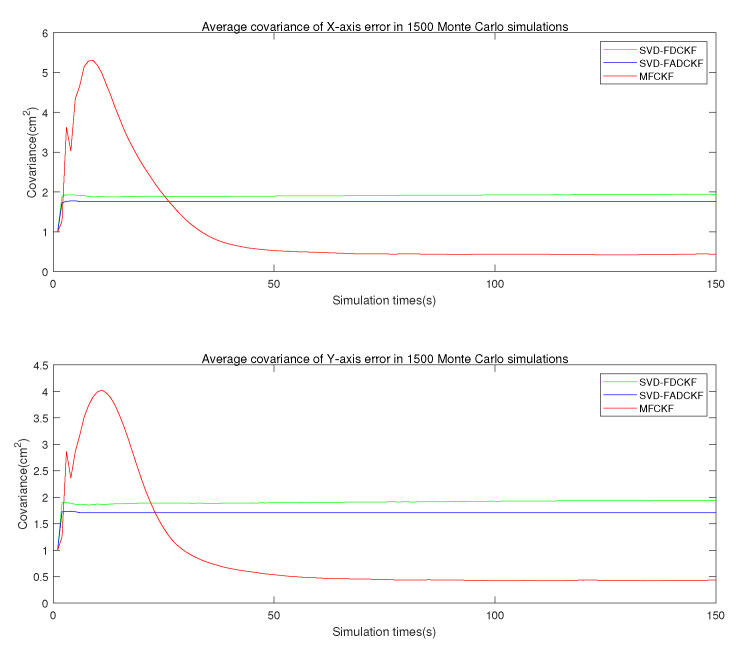
Average estimated error covariance in 1500 Monte Carlo experiments (3 algorithms).

Figure 10 shows the average estimation error covariance of each iteration in the 1500 Monte Carlo experiments. It can be seen that the proposed algorithm (MFCKF) has better performance during most of the simulation. However, identical to the state vector, the improved algorithm deteriorates in terms of initial overshoot and dynamic response performance, which may be caused by the increase in the order of the state vector. More efforts are needed to further address this issue.

**Figure 11:**
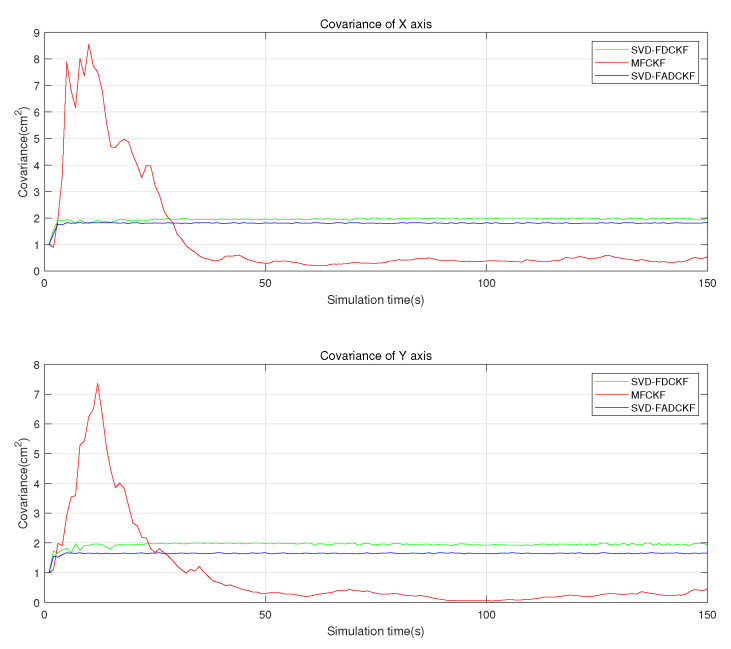
Estimated error covariance in in a selected simulation (3 algorithms).

Figure 11 shows the process of covariance converges in iteration. This is a randomly selected set from the 1500 Monte Carlo experiments. We can get a conclusion similar to Figure 10, the proposed algorithm has better performance in estimation error covariance after convergence but poor initial overshoot and dynamic response performance.

In addition, in the 1500 times Monte Carlo experiments, an average of 101.95 s per 150 s, the positioning error is contained in the error covariance curve, accounting for 67.97% of the total simulation time.

**Figure 12:**
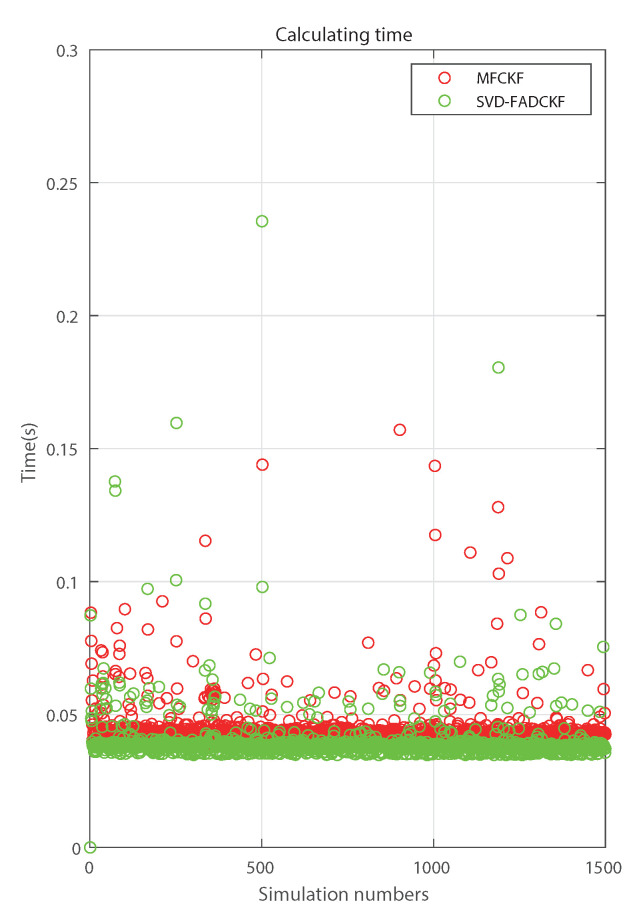
Comparison of simulation time of two algorithms.

Figure 12 shows that the calculation time of the proposed algorithm (SVD-MFDCKF) is slightly higher than that of the control group (SVD-FDCKF), which is ~11% (Table 5). This is basically consistent with the theoretical calculation complexity analysis shown in Table 2 (~12.11%). Nonetheless, the increased computational burden is considered to be worthwhile, considering the improvement in positioning accuracy 32.76%.

## 6. Conclusions

This study designed a set of solutions to solve the filtering divergence problem in applying the algorithm proposed by He et al. [25] in the high process noise environment. First of all, adaptive filtering was introduced to timely adjust the process noise covariance, which can help set *Q* optimally and thus increase the filtering accuracy, suppressing the filtering divergence. Subsequently, the state vector enhancement method is employed to enhance the system state vector and thus increase the traceability of the algorithm. Moreover, the state vector decomposition method is combined with the state vector enhancement method to simplify the enhanced state vector, thereby relieving the extra computational burden caused by the state enhancement method. The simplified system is demonstrated to have hardly increased computational burden according to comprehensive computational complexity analysis. Moreover, the overall positioning accuracy of the algorithm is improved. To verify the effectiveness of the algorithm, two sets of numerical simulations are carried out, which prove that the MFCKF proposed in this article has a significant improvement in positioning accuracy 32.76% compared to the original algorithm (SVD-FDCKF), although a small additional computational burden (11%) is introduced. Moreover, it can effectively perform accurate system state estimation in an environment with high process noise. Therefore, the algorithm proposed in this article achieves the expected goal.

Our future research will focus on the following two aspects:

- Transplanting the algorithm to a physical platform to verify the filtering performance of the proposed algorithm under actual conditions.
- Investigating the dynamic response performance of the proposed algorithm to further improve its performance in the highly dynamic environment.

## Figures and Tables

**Table 1:** Computational complexity analysis of three systems.

Step	Original System (5+5)	Augmented System (6+6)	Simplified Augment System (6+4)
Calculation of cubature points	450 flops 460 flops 200 flops	792 flops 844 flops 552 flops	508 flops 538 flops 300 flops
Time update	50 flops	72 flops	52 flops
Prediction of covariance update	260 flops	372 flops	270 flops
Measurement forecast	90 flops	132 flops	94 flops
Calculation of Kalman gain	1504 flops	2596 flops	1684 flops
Status update	30 flops	36 flops	30 flops
Covariance update	950 flops	1656 flops	1068 flops
Total	3994 flops	7052 flops	4544 flops

**Table 2:** Parameters of simulation.

Item	Parameters
Initial state	X coordinate: 4 (cm) Y coordinate: 4 (cm) Velocity: 5 (cm/s) Accelerate: 0 (cm/s${}^{2}$) Yaw angle: π/3 (${}^{\circ }$) Yaw angle changement: π/90 (${}^{\circ }$)
Simulation time	150 (/s)
Sampling time	T=1
Initial covariance	$P_{1}=\left[\begin{array}{cccccc} 1 & 0 & 0 & 0 & 0 & 0\\ 0 & 1 & 0 & 0 & 0 & 0\\ \;0 & 0 & 0.5 & 0 & 0 & 0\\ 0 & 0 & 0 & 0.1 & 0 & 0\\ \;0 & 0 & 0 & 0 & 0.15 & 0\\ \;0 & 0 & 0 & 0 & 0 & 0.001 \end{array}\right]$
Initial estimate	$X_{1}=\left[\begin{array}{c} 4\\ 4\\ 5\\ 0\\ \pi /3\\ \pi /90 \end{array}\right]$ + $\left[\begin{array}{c} N(0,1)\\ N(0,1)\\ N(0,0.5)\\ N(0,0.1)\\ N(0,0.15)\\ N(0,0.001) \end{array}\right]$
The driving matrix of process noise	$G_{k}=\left[\begin{array}{cccccc} 0 & 0 & 0 & 0 & 0 & 0\\ 0 & 0 & 0 & 0 & 0 & 0\\ \;0 & 0 & T & 0 & 0 & 0\\ 0 & 0 & 0 & 0 & 0 & 0\\ \;0 & 0 & 0 & 0 & \pi T/3 & 0\\ \;0 & 0 & 0 & 0 & 0 & \pi /3 \end{array}\right]$
Distribution of process noise $w_{k}$	$w_{k}=\left[\begin{array}{c} 0\\ 0\\ N(0,0.5)\\ 0\\ N(0,0.1)\\ N(0,0.01) \end{array}\right]$
Initial covariance of process noise $Q_{1}$	6-order diagonal identity matrix
Measurement noise of UWB	N(0,16)
White noise of gyro	N(0,0.05)
Constant drift of gyro	0.1°/h
White noise of accelerometer	N(0,0.001)
Constant bias of accelerometer	$10^{-3}$g
White noise of magnetometer	N(0,0.1)
White noise of odometer	N(0,0.1)

**Table 3:** Average positioning errors in 1500 Monte Carlo experiments.

Item	SVD-FDCKF	SVD-FADCK
Average positioning error (cm)	1.2937	1.1915

**Table 4:** Average positioning errors in 1500 Monte Carlo experiments.

Item	SVD-FDCKF	SVD-FADCK	MFCKF
Average positioning error (cm)	1.2937	1.1915	0.8698

**Table 5:** Average simulation time of two algorithms.

Item	SVD-FDCKF	MFDCK
Average calculation time (s)	0.0445	0.0398

## Data Availability

The data used in this study is available on request from the corresponding author.
